# Study of Hybrid Epoxy Resin Composites Reinforced with E-CR Glass Fiber/Polypropylene: Moisture Behavior, Mechanical Strength, and Acoustic Damage Mitigation Under Marine Aging Conditions

**DOI:** 10.3390/polym18212758

**Published:** 2026-11-05

**Authors:** Dunyansed Sarine Borges-Palomo, Carlos Rolando Ríos-Soberanis, Roberto Carlos Anaya-Ramírez, Alejandro Ortiz-Fernández, Emilio Pérez-Pacheco

**Affiliations:** 1 Centro de Investigación Científica de Yucatán, A. C. Unidad de Materiales, Calle 43 No. 130 x 32 y 34, Colonia Chuburná de Hidalgo, Merida 97205, Yucatan, Mexico; dunyansed.borges@estudiantes.cicy.mx (D.S.B.-P.); umat_ranaya@cicy.mx (R.C.A.-R.); 2 Tecnológico Nacional de México/Campus ITS_Calkiní. C. A. Bioprocesos, Av. Ah-Canul, Calkini 24900, Campeche, Mexico; aeortiz@itescam.edu.mx

**Keywords:** E-CR glass fiber, polypropylene fiber, epoxy composites, seawater aging, moisture diffusion, acoustic emission

## Abstract

The durability of fiber-reinforced epoxy composites in marine environments depends on the interaction among interfacial characteristics, moisture transport, and damage evolution. However, it remains poorly understood whether E-CR/PP hybridization merely provides a secondary reinforcing phase or also modifies moisture-transport and load-transfer mechanisms. This study investigated the effects of incorporating E-CR glass fibers and polypropylene (PP) fibers on moisture absorption, mechanical response, and damage development in an epoxy matrix. The reinforced composites were subjected to short-term seawater immersion for up to 30 days. FTIR-ATR, AFM, micromechanical testing, moisture absorption and diffusion analysis, flexural testing, and acoustic emission were combined to relate surface characteristics and interfacial behavior to moisture transport and mechanical performance. The neat epoxy matrix showed progressive moisture uptake and a 22.03% reduction in acoustic-wave propagation velocity, consistent with moisture-induced plasticization and reduced effective stiffness. The incorporation of 5 wt.% PP was associated with lower moisture uptake in composites containing equivalent E-CR contents and with a reduction in the estimated effective diffusion coefficient. The CPPV formulation, containing 50 wt.% E-CR and 5 wt.% PP, exhibited the lowest measured moisture uptake (1.20%) and effective diffusion coefficient (2.96 × 10^−12^ m^2^ s^−1^) among the formulations investigated during the early immersion period. After 30 days of seawater immersion, CPPV exhibited a residual flexural strength of approximately 600 MPa, compared with approximately 420 MPa for the corresponding 50 wt.% E-CR composite without PP (CFV). These findings indicate that PP does not act solely as a secondary reinforcing phase; rather, E-CR/PP hybridization modifies the effective moisture-transport environment and load-transfer response of the epoxy composite. Within the 30-day exposure period investigated, the hybrid architecture therefore provided improved resistance to moisture ingress and higher residual flexural strength. Longer-term exposure studies are required to establish whether these advantages persist under prolonged marine aging.

## 1. Introduction

Fiber-reinforced polymer matrix composites have emerged as a viable option for structural applications in marine environments because of their high strength-to-weight ratio, resistance to electrochemical corrosion, and ease of processing. Glass fiber/epoxy composites are particularly used in naval components, offshore structures, and elements subjected to prolonged exposure to moisture and salinity. However, corrosion resistance does not ensure stability against hygrothermal aging. Water can penetrate the epoxy network, interact with polar groups and free volume, induce plasticization and swelling, and reach the fiber–matrix interface. This may lead to loss of interfacial adhesion, microcracking, and fiber–matrix interfacial debonding. Therefore, marine durability depends on interactions among the matrix, reinforcement, interface, and moisture transport pathways, rather than solely on the composite’s initial strength [1,2,3].

Reports indicate that these processes can cause significant mechanical degradation. Rubio-González et al. [4] investigated glass fiber/epoxy laminated composites manufactured by resin transfer molding and aged in artificial seawater at 70 °C for 1464 h. They then performed low-velocity impact testing, ultrasonic C-scan analysis, electron microscopy, and computed tomography. Aging reduced the maximum impact force by about 40% and significantly increased the damaged area, associated with matrix plasticization, fiber–matrix debonding, and delamination. Kirar and Demircan [5] studied S2-glass fiber/epoxy composites manufactured by vacuum-assisted infusion and aged in artificial seawater for 4, 8, and 12 months. Compression tests in both directions at different loading rates showed strength reductions of up to 23.48%, confirming that marine exposure affects both effective stiffness and rate-dependent response. Yalçınkaya et al. [6] compared glass fiber-reinforced polymer (GFRP) and carbon fiber-reinforced polymer (CFRP) laminates manufactured by hand lay-up and hot pressing. After aging in Aegean seawater for 30 and 60 days, they evaluated moisture absorption, Charpy impact behavior, three-point flexural response, and microscopic features. They concluded that GFRP shows greater moisture uptake and is more mechanically sensitive than CFRP.

Other studies have demonstrated that matrix and interface composition play a key role in degradation. Wang et al. [7] compared three epoxy systems cured at different temperatures and a vinyl ester resin, then subjected them to seawater immersion at 25, 40, and 60 °C. Through diffusion analysis and mechanical property evaluation, they showed that higher curing temperatures reduce equilibrium moisture uptake by decreasing the availability of polar groups, thereby improving mechanical property retention. José-Trujillo et al. [8] aged glass fiber/epoxy and glass fiber/vinyl ester laminates to saturation in accordance with ASTM D5229. They then conducted uniaxial tensile, compression, flexural, and interlaminar shear tests and combined the results with neural network analysis. They found that the epoxy matrix loses significant strength, particularly under compression and interlaminar shear, and that time-dependent damage evolution can be modeled as a function of immersion time. Alsaadi et al. [9] compared basalt fiber/epoxy and basalt fiber/Elium composites exposed to seawater at 45 °C for 45 and 90 days. Tensile and flexural tests, along with electron microscopy, assessed resin and fiber surface degradation and revealed greater deterioration in flexural properties. These findings confirm that the interfacial region is particularly sensitive to the marine environment.

Controlling the fiber–matrix interface is therefore an important strategy for improving the resistance of composite materials to environmental aging. Sidharth et al. [10] investigated sized and unsized glass and basalt fibers in thermoplastic and epoxy matrices using vacuum-assisted infusion, single-fiber push-out testing, and nanoindentation. The authors showed that sizing affects both interfacial shear strength and interphase thickness, and that its influence varies with the specific compatibility between the fiber and the matrix. These findings are particularly relevant to E-CR glass fibers containing silane coupling agents, because the same behavior does not directly extend to chemically nonpolar reinforcements such as polypropylene. Beyond modifying the fiber–matrix interface, researchers have also investigated hybrid reinforcement strategies to control moisture uptake while maintaining mechanical performance. Huang et al. [11] developed hybrid epoxy systems reinforced with kenaf, flax, and glass fibers using hand lay-up and vacuum bagging techniques. They evaluated tensile, flexural, and moisture-absorption behavior in distilled water and artificial seawater. Hybridization reduced moisture uptake and improved mechanical properties compared with the single-fiber system, confirming that hybrid architecture can modify both moisture transport and load transfer.

Hybridization with PP presents a distinct scenario compared with natural or carbon fibers. E-CR glass fiber provides high stiffness and a sizing-functionalized surface, whereas PP has a lower modulus, greater deformability, and hydrophobic character, although it also exhibits low surface energy and lower chemical affinity for epoxy resins. Incorporating PP could modify effective moisture-transport pathways through the matrix and interfacial regions, thereby affecting water-diffusion kinetics. Wang et al. [7] investigated GFRP bars with a PP matrix exposed to saline and saline-alkaline environments and analyzed water absorption, DMA, and interlaminar shear strength. The low equilibrium moisture content and interlaminar strength retention demonstrated PP’s potential for marine environments, although interfacial debonding and surface defects were also detected, creating major pathways for water ingress. These studies show that the hydrophobicity of either the matrix or the reinforcement does not diminish the importance of the interface [12,13].

The macroscopic response alone is also insufficient to determine the extent of damage. Cunha et al. [14] manufactured hybrid laminates reinforced with carbon, basalt, aramid, and glass fibers and subjected them to flexural testing at different loading rates while simultaneously recording acoustic emissions. Signal classification correlated acoustic activity with internal fracture mechanisms and demonstrated the technique’s potential for in-service damage monitoring. In GFRP-concrete composites, Svetlik-Haley and Karbhari [15] combined mechanical testing, acoustic emission, historical index, severity, and the sentry function to investigate stress transfer and interfacial deterioration, showing that acoustic emission can identify changes associated with chemical adhesion, mechanical interlocking, friction, and slip. Together with recent analyses of interfacial chemistry and damage monitoring [16], these studies indicate that acoustic emission provides greater diagnostic value when correlated with independent information on the matrix, fibers, and interface.

Despite these advances, the relationship between E-CR/PP hybridization, seawater transport, and the micromechanical evolution of damage in epoxy matrices remains poorly understood. The literature has extensively examined conventional GFRP, hybrid systems containing natural fibers, thermoplastic matrices, and interface modification strategies. However, few studies have simultaneously considered the surface topography and chemistry of E-CR and PP, single-filament response, moisture absorption and diffusion, residual mechanical properties, and acoustic emission to establish relationships among composition, interface characteristics, transport behavior, and damage evolution. This knowledge gap limits the ability to determine whether PP acts merely as a secondary reinforcing phase or whether it effectively modifies moisture pathways and load-transfer mechanisms in E-CR glass fiber/epoxy composites.

This study arises from the need to establish mechanism-based design criteria rather than relying solely on residual strength. Its novelty lies in integrating surface and chemical characterization, micromechanical analysis, diffusion assessment, and acoustic emission monitoring to investigate a hybrid composite of E-CR glass fibers, polypropylene fibers, and epoxy resin exposed to marine environments. It is proposed that the controlled incorporation of PP modifies both effective water-absorption kinetics and load transfer because of its hydrophobic nature, greater deformability, and distinct interfacial compatibility compared with E-CR glass. This study aims to evaluate the effects of hybridization with E-CR glass fibers and polypropylene fibers on moisture transport, mechanical response, and damage mechanisms in an epoxy matrix subjected to seawater immersion, using Fourier transform infrared spectroscopy (FTIR) and atomic force microscopy (AFM) characterization, mechanical testing, absorption and diffusion analysis, and acoustic emission (AE) monitoring. From an application perspective, such hybrid composites could be considered for secondary panels and protective components in marine vessels, where reduced moisture ingress and retained mechanical performance under saline exposure are desirable. A second potential application is in non-critical components of offshore structures exposed to splash-zone or humid saline conditions, such as protective covers, enclosures, and secondary structural panels. These potential applications require further validation through long-term marine aging, fatigue, impact, and component-scale testing before practical implementation.

## 2. Materials and Methods

### 2.1. Materials

A commercial DER™ 331 epoxy resin based on diglycidyl ether of bisphenol A (DGEBA) was used as the polymer matrix, with Ancamine^®^ 1784 as the curing agent. These components formed the thermosetting system used to manufacture the neat matrix and composite materials.

Continuous E-CR glass fibers were used as the primary reinforcement and were supplied as commercial OptiSpray™ F-2400 roving (Poliformas Plásticas, Ciudad Nezahualcoyotl, Mexico). The filaments ranged from 18 to 24 μm in diameter and were treated with a sizing containing a silane coupling agent. Continuous polypropylene (PP) fibers in roving form, purchased from Poliformas Plásticas (Ciudad Nezahualcoyotl, Mexico), were used as the secondary reinforcement. The PP filaments ranged from 15 to 19 μm in diameter.

Based on these materials, one neat epoxy matrix and four reinforced compositions were evaluated. Seawater for the aging tests was prepared in accordance with ASTM D1141-98 [17] using analytical-grade salts dissolved in distilled water.

### 2.2. Matrix Preparation and Composite Manufacturing

The thermosetting matrix was prepared using DER™ 331 epoxy resin and Ancamine^®^ 1784 curing agent. The composites were manufactured by casting the epoxy matrix with continuous E-CR glass and polypropylene (PP) rovings positioned longitudinally in the molds. The rovings were arranged nominally parallel to the longitudinal axis of the specimens and, consequently, to the primary loading direction used during mechanical characterization.

Four reinforced formulations with different E-CR glass and PP fiber mass fractions were prepared. The composition of each formulation is summarized in Table 1. MFV contained 25 wt.% E-CR glass fiber and 75 wt.% epoxy matrix, whereas CFV contained 50 wt.% E-CR glass fiber and 50 wt.% epoxy matrix. For the hybrid formulations, PP fibers represented 5 wt.% of the total composite mass, while the E-CR glass fiber fraction remained the same as in the corresponding non-hybrid formulation. Accordingly, MPPV contained 25 wt.% E-CR glass fiber, 5 wt.% PP fiber, and 70 wt.% epoxy matrix, whereas CPPV contained 50 wt.% E-CR glass fiber, 5 wt.% PP fiber, and 45 wt.% epoxy matrix. Thus, the incorporation of PP was achieved by reducing the epoxy-matrix fraction while maintaining the nominal E-CR glass fiber content. Neat epoxy specimens containing no reinforcement were also manufactured and used as reference material.

The E-CR and PP rovings were positioned longitudinally prior to matrix addition to maintain filament continuity and nominal longitudinal orientation. The reinforcements were subsequently impregnated with the epoxy mixture, and the system was formed by casting into molds with the geometry required for each test. The original experimental program did not include quantitative measurements of void content, fiber-orientation angle, or spatial fiber distribution. Therefore, the reported fiber orientation refers to the nominal orientation imposed during specimen fabrication rather than to a quantitative microstructural measurement of the cured composites. Figure 1 shows the sequence of processes for the manufacture of a polymer composite.

After fabrication, the specimens were maintained at 25 °C for 48 h to allow initial curing of the epoxy matrix. They were then removed from the molds and conditioned according to the corresponding test procedure. This manufacturing methodology was applied to the MFV, CFV, MPPV, and CPPV formulations to enable subsequent comparison of the effects of E-CR content, PP incorporation, and seawater exposure on the composite response.

### 2.3. Atomic Force Microscopy

The surfaces of the E-CR and PP fibers were analyzed using a Bruker Innova IRIS AFM (Santa Barbara, CA, USA) operated in tapping mode. A silicon tip with a 10 nm radius and a cantilever with a spring constant of 0.06 N/m was used. The interaction force, approximately 10^−8^ N, was adjusted to each fiber’s characteristics to minimize surface modification during scanning.

### 2.4. Fourier Transform Infrared Spectroscopy with Attenuated Total Reflectance (FTIR-ATR)

The chemical composition of the E-CR fibers, PP fibers, and epoxy matrix was evaluated by Fourier transform infrared spectroscopy with attenuated total reflectance. A Nicolet 8700 spectrometer (Thermo Scientific, Madison, WI, USA) equipped with a 1.8 mm diameter zinc selenide (ZnSe) ATR crystal collected spectra from 4000 to 500 cm^−1^. The fibers and epoxy matrix were analyzed in their original state, both before and after exposure to seawater.

### 2.5. Micromechanical Characterization of the Fiber–Matrix Interface

To analyze the E-CR glass fiber-epoxy resin and polypropylene fiber-epoxy resin interfaces separately, Dog-bone specimens containing a single fiber filament aligned with the loading direction were prepared in accordance with ASTM D638 [18] (Figure 2) and tested; subsequently, stress–strain curves were recorded to determine ultimate tensile strength, maximum strain, and elastic modulus. Individual filaments were manually separated from the corresponding E-CR glass and PP rovings and positioned longitudinally along the centerline of the specimen mold before casting the epoxy resin. The embedded filament length was 27 mm. After curing, ten independent replicate specimens were tested for each fiber-epoxy system. A systematic post-test fractographic examination was not performed; therefore, specific failure mechanisms, such as fiber fracture, fiber–matrix debonding, or interfacial sliding, could not be conclusively distinguished from the available experimental evidence. Uniaxial tensile tests were performed using the Mini Shimadzu AGS-X system (Shimadzu Corporation, Kyoto, Japan) equipped with a 1000 N load cell at a speed of 1 mm min^−1^. Simultaneously, recordings of the mechanical response and acoustic emission activity were carried out.

### 2.6. Acoustic Emission

Before tensile testing, wave propagation was verified on each sample by using the Hsu-Nielsen method, with the controlled breakage of a graphite pencil lead at 45° used as a reproducible acoustic source. The propagation velocity was determined for the neat epoxy matrix and for systems containing a single E-CR or PP filament, providing a reference for subsequent analysis.

During mechanical testing, acoustic emission parameters, including amplitude and energy, were recorded synchronously with the stress–strain response. AE signals were obtained during mechanical tests using MISTRAS II PCI-2 data acquisition equipment (Physical Acoustics Corporation, Princeton Junction, NJ, USA), with a sampling frequency of 8 MHz, 40 dB preamplification, and a 30 dB threshold. To detect acoustic signals, two small piezoelectric sensors were placed on the sample surface.

### 2.7. Marine Aging and Moisture Absorption

The neat epoxy matrix and reinforced formulations were continuously immersed in seawater at approximately 25 °C. Moisture absorption of the neat epoxy matrix was evaluated according to ASTM D570 [19], whereas the reinforced composites were evaluated according to ASTM D5229/D5229M-20 [20].

The gravimetric monitoring schedule was selected to characterize different stages of the moisture-uptake process. Shorter sampling intervals were used during the initial immersion period, when the largest changes in specimen mass were expected, allowing the initial moisture-transport behavior to be adequately characterized. At longer exposure times, sampling intervals increased progressively as the rate of mass change decreased and the specimens approached gravimetric equilibrium. For the neat epoxy matrix, monitoring was extended to approximately 365 days to characterize both the initial absorption stage and the longer-term evolution toward equilibrium.

For the reinforced formulations (MFV, CFV, MPPV, and CPPV), moisture uptake was monitored for 30 days. This exposure period was selected to provide a common short-term marine-aging condition for comparing the effects of E-CR glass-fiber content and PP hybridization on moisture transport. The same 30-day exposure period was subsequently used for the mechanical characterization of the reinforced formulations, enabling moisture uptake to be related to the residual mechanical response. Therefore, the 30-day period was used as a comparative short-term conditioning interval and was not assumed to represent complete moisture saturation of the composites.

At each measurement time, specimens were individually removed from the immersion medium, and absorbent material gently removed the water adhering to their external surfaces. Care was taken to remove only superficial water and to avoid prolonged drying that could remove absorbed moisture from the specimens. Immediately after removing surface water, each specimen was weighed on a balance with a resolution of 0.0001 g. After recording the mass, specimens intended for continued monitoring were immediately returned to the immersion medium. The same procedure was consistently applied at each measurement interval. Moisture content was calculated according to the gravimetric relationship specified in these test methods, as expressed in Equation (1):

(1) $$M_{t}\left(\%\right)=\frac{m_{t}-m_{0}}{m_{0}}\times 100$$

where *M_t_* is the moisture absorption at time *t* (%), *m_t_* is the mass of the specimen after an immersion time *t*, and *m*_0_ is the initial mass of the specimen in the dry state.

### 2.8. Flexural Characterization Before and After Seawater Exposure

Flexural mechanical characterization of the epoxy resin was carried out to observe changes in strength upon the addition of fibers as reinforcement in dried and after-water exposure conditions. Test specimens for flexural characterization were prepared in accordance with ASTM D790 [21] (Figure 3). All samples (epoxy resin and composites) were tested after curing under dry conditions and after 30 days of seawater immersion.

The mechanical response of the neat matrix and composites was evaluated by three-point flexural testing using an 80 mm support span, a crosshead speed of 5 mm min^−1^, and eight specimens per condition. Flexural strength and flexural modulus were determined from the load–displacement curves. Flexural strength was calculated according to ASTM D790 [21] using Equation (2):

(2) $$\sigma_{f}=\frac{3\mathrm{FL}}{2ba^{2}}$$

where *F* is the applied load, *L* is the support span, *b* is the width, and *a* is the thickness.

### 2.9. Statistical Analysis

Statistical analysis was applied to quantitative measurements for which independent experimental replicates were available. The number of specimens or independent measurements used for each quantitative test is specified in the corresponding experimental subsection. Micromechanical characterization was performed using ten independent specimens for each fiber-epoxy system (*n* = 10), whereas flexural characterization was performed using eight independent specimens per experimental condition (*n* = 8). The number of specimens used for moisture-absorption measurements is also specified in Section 2.7.

Results obtained from replicated quantitative measurements are reported as mean ± standard deviation (SD). Individual experimental measurements were retained in the statistical analysis unless a value could be demonstrated to result from an identifiable experimental or measurement error. AFM and FTIR-ATR analyses were used as complementary surface and chemical characterization techniques, respectively, and their results are therefore presented as representative characterization measurements rather than as population-level statistical data.

## 3. Results and Discussion

### 3.1. Atomic Force Microscopy (AFM)

Figure 4 shows AFM images of the surfaces of E-CR glass and polypropylene (PP) fibers. Topographic analysis revealed morphological differences between the two reinforcement types. The E-CR fibers exhibited a relatively uniform surface with isolated protrusions, whereas the PP fibers showed greater topographic variability and higher surface roughness. The E-CR fibers analyzed ranged from 18 to 24 μm in diameter and were surface-treated with a silane coupling agent. In contrast, the PP fibers ranged from 15 to 19 μm in diameter.

The AFM results revealed two distinct surface characteristics. The E-CR surface was more homogeneous, with discrete irregularities, whereas the PP surface exhibited greater topographic variability. The protrusions observed on E-CR may be attributed to the sizing applied during fiber manufacturing.

These topographic differences are critical to understanding the micromechanical behavior of the E-CR/epoxy and PP/epoxy systems because stress transfer in fiber-reinforced composites depends on both surface chemistry and morphology within the contact region. Fiber topography can modify the effective contact area, matrix wetting, and mechanical interlocking, and can also affect interfacial strength. Recent studies suggest considering both surface roughness and sizing chemistry when analyzing load transfer between the fiber and the matrix [10].

For the E-CR fibers, the AFM-detected protrusions are consistent with a surface modified during industrial fiber processing. This interpretation is supported by the material specification used in this study, which indicates that Advantex^®^ fibers contain a sizing system with a silane coupling agent. Sizing is particularly relevant in glass fiber/epoxy composites because it protects the filaments during processing and can influence chemical compatibility, wetting, and stress transfer at the interface [10].

The greater topographic heterogeneity observed in PP suggests a different interfacial mechanism. A rougher surface may increase contact with the resin and enhance mechanical interlocking after curing. However, greater roughness does not automatically ensure superior interfacial adhesion. Effective adhesion results from the combined effects of topography, surface energy, wettability, and the chemical composition of both phases. This distinction matters because FTIR results from the same study showed clear chemical differences between the reinforcements. E-CR fibers contain more polar groups, whereas the PP spectrum is dominated by predominantly nonpolar C-H bond vibrations. PP’s higher roughness may enhance mechanical interlocking at the interface, but it alone does not ensure chemical interactions comparable to those between a silane-treated glass fiber surface and the epoxy matrix.

This interpretation suggests two distinct interfacial mechanisms. For E-CR/epoxy, load transfer likely depends on the interaction between surface topography and sizing chemistry. In contrast, for PP/epoxy, physical contact and mechanical interlocking are likely to be the dominant mechanisms because PP is less polar. This distinction aligns with reports in the literature indicating that improving interfacial strength requires optimizing both surface roughness and physicochemical interactions [10,16].

Specimens with a single embedded filament showed different acoustic propagation velocities in the E-CR/epoxy and PP/epoxy systems, showing that each fiber type alters the system’s effective elastic response differently. However, do not assume a direct causal relationship between surface roughness and acoustic velocity. AFM characterizes surface topography before composite formation, whereas the acoustic response of the embedded-filament specimen reflects the combined contributions of the fiber, matrix, interface, and any existing discontinuities.

The interface may act as a moisture transport pathway when the fibers are not fully wetted or when interfacial discontinuities are present. In the absorption experiments in this study, the glass fiber-reinforced materials exhibited behavior consistent with additional transport pathways through the fiber–matrix interfacial regions. In contrast, the hybrid formulation containing 50% E-CR and 5% PP exhibited the lowest effective diffusion coefficient among all compositions analyzed. The PP topography observed by AFM does not indicate that this fiber acts as a water barrier; however, when considered together with the hydrophobic chemical character identified by FTIR, it provides a microstructural basis for understanding the lower diffusion observed in the hybrid formulation.

Therefore, the AFM results show that E-CR/PP hybridization introduces two reinforcement surfaces with distinct topographic and chemical characteristics, which may generate different adhesion mechanisms with the epoxy matrix. This finding provides a microstructural basis for understanding the material response at larger scales. The E-CR surface exhibits a relatively uniform topography with features associated with the silane-based surface treatment, whereas PP shows greater topographic heterogeneity and predominantly nonpolar chemistry. These characteristics may alter stress transfer, moisture ingress pathways, and damage evolution under loading. However, quantitatively demonstrating these relationships requires correlating the AFM results with FTIR, micromechanical, absorption/diffusion, and acoustic emission data. This integrated analysis constitutes the mechanistic core of the present study.

### 3.2. Fourier Transform Infrared Spectroscopy-Attenuated Total Reflectance (FTIR-ATR)

Figure 5 presents the FTIR-ATR spectra of the E-CR glass and polypropylene (PP) fibers. The PP spectrum showed prominent bands at approximately 2950 and 2870 cm^−1^, together with signals near 1453 and 1370 cm^−1^. Additional lower-intensity bands were observed at approximately 1164, 1092, 799, and 714 cm^−1^. The E-CR glass-fiber spectrum exhibited signals in the high-wavenumber region, together with bands at approximately 2880, 1740, 884, and 730 cm^−1^. Weak features observed in the 2300–2400 cm^−1^ region were not used for structural assignment because contributions from atmospheric species or instrumental background cannot be excluded.

For PP, the bands near 2950 and 2870 cm^−1^ are consistent with aliphatic C-H stretching vibrations, whereas the signals near 1453 and 1370 cm^−1^ are associated with CH_2_/CH_3_ deformation modes characteristic of polypropylene. The lower-wavenumber bands are associated with skeletal and C-H vibrational modes of the polymer. Overall, the spectrum is consistent with the predominantly hydrocarbon and nonpolar chemical structure of PP.

The E-CR glass-fiber spectrum showed a different spectral profile. Features in the high-wavenumber region are consistent with hydroxyl-containing species at or near the glass surface; however, the present ATR spectrum alone cannot unequivocally assign them to specific silanol or adsorbed-water species. The bands observed at lower wavenumbers are consistent with contributions from the silicate-based glass surface and its commercial sizing. In addition, the signals near 2880 and 1740 cm^−1^ may correspond to organic components of the sizing system, including aliphatic and carbonyl-containing species, respectively.

Therefore, the FTIR-ATR results support chemically distinct surfaces for the two reinforcement types: PP shows a predominantly hydrocarbon character, whereas E-CR glass fiber shows contributions from the inorganic glass surface and its organic sizing. These assignments are interpreted as qualitative spectral evidence and not as unequivocal identification of individual chemical species.

Comparison of the two spectra indicates two chemically distinct interfacial environments in the E-CR/PP/epoxy hybrid system. The E-CR glass fiber exhibits contributions from the inorganic glass surface and the commercial sizing, whereas PP is dominated by hydrocarbon-related vibrational bands. These differences are expected to influence the interaction of each reinforcement with the epoxy matrix. However, FTIR-ATR results alone do not provide a direct, quantitative measure of fiber–matrix adhesion. Interfacial behavior also depends on surface topography, wettability, mechanical interlocking, and sizing-layer characteristics. Therefore, interpret the chemical differences identified by FTIR in conjunction with the AFM, micromechanical, moisture-absorption, and acoustic-emission results.

The higher surface polarity of glass may promote interactions with water molecules and facilitate interfacial hydration processes, whereas the nonpolar character of PP reduces its direct affinity for water. Nevertheless, recent studies on GF/PP composites indicate that PP’s hydrophobicity does not prevent moisture transport, because water ingress tends to concentrate at defects, interfaces, and surface discontinuities [7]. Therefore, the effect of PP on composite durability should not be interpreted solely as a hydrophobic barrier, but also as a chemical and structural modification of effective moisture-transport pathways.

Recent studies provide strong evidence for the importance of sizing. Using single-fiber push-out testing and nanoindentation, Sidharth et al. [10] showed that sizing modifies interfacial shear strength and interphase thickness in glass fiber systems. Colombo et al. [16] emphasized that fiber surface chemistry influences load transfer and damage development in GFRP, while Liu et al. [22] showed that controlled sizing modification can increase surface roughness, compatibility, and interfacial energy dissipation. These observations suggest that the FTIR signals of E-CR glass fiber represent not only a chemical fingerprint of the glass surface but also evidence of a functionalized surface that may directly affect its subsequent mechanical behavior.

The FTIR results show that E-CR and PP fibers introduce two chemically distinct surfaces into the epoxy matrix. E-CR combines a silicate network with polar species and a silane-based surface treatment, whereas PP exhibits a predominantly hydrocarbon and nonpolar structure. This duality provides a physicochemical basis for understanding the differences subsequently observed in wetting, stress transfer, and moisture transport. E-CR/PP hybridization should therefore not be regarded merely as the combination of two reinforcements with different elastic moduli, but as the incorporation of two distinct interface types with different chemical affinities for both the matrix and the marine environment.

### 3.3. Effect of Seawater on the Matrix

Figure 6 shows the FTIR spectra of the epoxy matrix before and after seawater exposure. The spectrum of the unaged resin exhibited the characteristic bands of the epoxy system: a broad band between 3600 and 3000 cm^−1^, mainly associated with O-H vibrations; signals between 2915 and 2840 cm^−1^, corresponding to aliphatic C-H stretching; bands around 1601 and 1502 cm^−1^, associated with aromatic rings; and peaks in the 1236–1010 cm^−1^ region, related to C-O-C ether bonds. A band around 922 cm^−1^, attributed to residual epoxy groups, was also identified.

The spectral changes suggest that the aqueous environment affected the epoxy network’s local chemical environment. However, a decrease in FTIR intensity does not constitute definitive evidence of bond cleavage or irreversible chemical degradation, because the apparent ATR intensity is also influenced by sample-crystal contact, effective penetration depth, moisture content, and surface heterogeneity. Therefore, the most accurate interpretation is that seawater exposure induced changes in the matrix’s molecular environment consistent with moisture uptake and plasticization.

Wang et al. [7], in their evaluation of several polymer matrices exposed to seawater, showed that moisture uptake depends strongly on the concentration of polar groups and the degree of matrix crosslinking; they also linked increased water content to a progressive loss of mechanical properties attributed to plasticization. Rubio-González et al. [4] attributed the degradation of seawater-aged glass/epoxy laminates to moisture absorption and the resulting softening of the epoxy matrix. These studies support the mechanism proposed for DER 331 resin.

Gravimetric evidence indicates that water was incorporated into the matrix. As shown in Table 2, the average mass increased from 25.2 to 25.7 g after immersion. Based on these values, the calculated relative increase was approximately 1.90%, which agrees with the experimental value of nearly 2%. At the same time, the wave propagation velocity decreased from 1645.16 to 1282.79 m s^−1^, corresponding to a 22.03% reduction.

The coexistence of a moderate mass increase and a reduction of more than one-fifth in wave velocity is one of the most notable findings of this stage. The mass increase indicates water-molecule incorporation, whereas the decrease in propagation velocity suggests a significant change in the material’s effective elastic response. In a homogeneous polymeric solid, mechanical wave propagation velocity depends on its effective stiffness and density. Because the observed mass variation was small compared with the reduction in wave velocity, the acoustic change is unlikely to result solely from the density increase associated with moisture uptake. Instead, the result primarily indicates a decrease in the epoxy network’s effective stiffness.

This behavior is consistent with water-induced plasticization. Water molecules can penetrate the thermoset network’s free volume and form hydrogen bonds with polar groups within the epoxy structure. These interactions reduce effective intermolecular forces between chain segments and increase segmental mobility, thereby decreasing stiffness and, consequently, elastic-wave propagation velocity. Wang et al. [7] observed that epoxy matrices with higher moisture uptake retained fewer mechanical properties after marine aging. Alsaadi et al. [9] also associated aging in reinforced epoxy systems with moisture absorption, localized matrix degradation, and preferential deterioration of resin-dominated properties.

The magnitude of the reduction in wave velocity indicates that the acoustic response could serve as a sensitive parameter for detecting moisture-induced changes before the matrix exhibits visually evident deterioration. However, these results should be interpreted as indicators of changes in the medium’s stiffness or continuity rather than as an exact measure of the extent of chemical degradation. Microvoids, microcracking, differential swelling, and heterogeneities generated during conditioning may also influence wave velocity.

The time-dependent evolution of moisture absorption provides additional insight into this mechanism. As shown in Figure 7, the epoxy matrix showed a rapid mass increase during the initial exposure stage. Moisture uptake increased from 0.15% on the first day to 1.37% after 7 days, then reached 1.49% after 15 days and 1.65% after 30 days. Over longer exposure periods, absorption continued to increase, reaching 4.12% after 182 days and 4.28% after 364 days. At the same time, the swelling rate decreased from approximately 0.05–0.056 g day^−1^ during the first few days to 0.015 g day^−1^ after 30 days, 0.006 g day^−1^ after 182 days, and ultimately 0.003 g day^−1^ after 364 days.

The curve clearly shows two regimes. During the first stage, a pronounced concentration gradient exists between the marine environment and the matrix interior, promoting rapid water ingress. Subsequently, as the internal moisture content increases, the concentration gradient decreases and the absorption rate declines. The value of 4.28% after approximately one year, together with a swelling rate approaching zero, suggests that the specimen is approaching hygroscopic equilibrium under the experimental conditions and geometry considered. This does not necessarily imply that all physicochemical processes have reached equilibrium, but rather that the gravimetric variation has become small.

The shape of this curve indicates that the initial kinetics are primarily diffusion-controlled. However, the long-term results suggest that ideal Fickian behavior should not be assumed automatically. Moisture uptake in epoxy systems may arise from free water occupying available free volume and bound water associated with polar groups within the network. Wang et al. [7] showed that polar-group concentration and curing temperature affect both equilibrium moisture uptake and the diffusion mechanism in epoxy resins. Yalçınkaya et al. [6], José-Trujillo et al. [8], and Fu et al. [23] highlighted the close relationship among moisture absorption, plasticization, swelling, and loss of mechanical properties in thermosetting matrices and composites exposed to humid or saline environments.

The results support the following aging sequence: water initially penetrates the matrix, driven by the concentration gradient, and occupies regions of free volume. Subsequently, a fraction of these water molecules associates with polar groups in the epoxy network, primarily hydroxyl and ether groups. These interactions increase segmental mobility, induce plasticization and swelling, and reduce the material’s effective stiffness. With prolonged exposure, additional processes may occur, including structural relaxation, microcracking, or localized hydrolysis.

The results show that the epoxy matrix is vulnerable to prolonged seawater exposure. The approximately 1.9% mass gain after immersion was accompanied by a 22.03% reduction in wave propagation velocity. Long-term monitoring also showed moisture uptake of approximately 4.28% after 364 days. The convergence of the gravimetric, spectroscopic, and acoustic responses supports an aging mechanism initiated by water absorption and plasticization of the epoxy network.

### 3.4. Moisture Absorption and Effective Diffusion

Figure 7 also shows the evolution of moisture absorption in the neat epoxy matrix. The most rapid mass increase occurred during the first seven days of immersion, rising from 0.15% after 1 day to 1.37% after 7 days. Thereafter, the absorption rate decreased progressively: mass gain was 1.49% after 15 days, 1.65% after 30 days, 4.12% after 182 days, and 4.28% after 364 days of exposure. Likewise, the swelling rate increased from 0.044 to 0.056 g day^−1^ during the initial stage, then decreased to 0.028 g day^−1^ after 15 days, 0.015 g day^−1^ after 30 days, and finally 0.003 g day^−1^ at the end of the monitoring period.

The curve shows an initial stage with a high driving force for water transport, followed by a gradual decrease in the absorption rate as the concentration gradient between the external medium and the matrix interior diminishes. The swelling rate approaching zero after prolonged exposure suggests that the system is approaching gravimetric equilibrium, although this does not guarantee that all physicochemical processes associated with aging have also reached equilibrium. In epoxy matrices, water transport may involve diffusion through free volume, interaction with polar groups, and progressive relaxation of the polymer network. For this reason, a curve that appears to approach saturation should not necessarily be regarded as evidence of ideal Fickian behavior throughout the entire exposure period.

This behavior is consistent with reports in the literature. Sindu and Sasmal [24] demonstrated that moisture and salinity jointly affect the properties of epoxy systems used in offshore applications, linking water uptake to changes in both the matrix and interfacial regions. Buckingham et al. [12] indicated that moisture in GFRP can enter through diffusion within the matrix and by preferential transport along interfaces and microstructural discontinuities. These observations agree with the epoxy matrix behavior in this study, where the absorption rate was highest during the first few days and then gradually decreased as internal moisture content increased.

After 15 days of immersion, the reinforced formulations showed marked differences in mass gain but not in swelling rate. As shown in Figure 7, MFV exhibited the highest moisture uptake, reaching 1.91%, followed by MPPV at 1.43%, CFV at 1.33%, and CPPV at 1.20%. The corresponding swelling rates were 0.034, 0.033, 0.032, and 0.031 g day^−1^, respectively. These results show a consistent reduction in moisture absorption with changes in reinforcement composition, although the differences in swelling rate after 15 days were relatively small.

Comparing formulations with the same amount of E-CR glass fiber more clearly indicates the effect of PP addition. From MFV to MPPV, mass gain decreased from 1.91% to 1.43%, a reduction of approximately 24.7%. Similarly, the transition from CFV to CPPV reduced moisture absorption from 1.3% to 1.2%, representing a decrease of approximately 10.1%. These results show that 5% PP was associated with reduced moisture uptake at both E-CR glass fiber contents. However, the magnitude of this effect varied by composition, with a greater reduction in the system with the lower glass fiber fraction.

Two mechanisms can explain this behavior. First, PP is predominantly nonpolar and has low affinity for water; therefore, its incorporation locally reduces the amount of material that can interact with water molecules. Second, introducing a secondary fibrous phase alters matrix connectivity and the geometry of available transport pathways. However, the available data do not support the conclusion that PP forms a continuous barrier or directly blocks diffusion pathways. A more accurate interpretation is that hybridization modifies effective moisture transport through combined changes in composition, tortuosity, and interfacial structure.

Adding fibers may create additional transport pathways through the fiber–matrix interfacial regions. This is consistent with recent studies identifying interfaces as critical regions for moisture transport in polymer composites. De Leon et al. [25] demonstrated that hygrothermal aging directly affects the interfacial behavior of polymer composites, whereas Christ et al. [26], using micromechanical testing, observed that moisture can reduce interfacial strength by penetrating the fiber–matrix region. Increasing the reinforcement content may therefore produce two opposing effects: it may increase the tortuosity of transport through the matrix, while also increasing the interfacial area available for water ingress.

Table 3 shows that the competition between these mechanisms depends on composition. The estimated effective diffusion coefficients were 3.04 × 10^−12^ m^2^ s^−1^ for the neat matrix, 3.13 × 10^−12^ m^2^ s^−1^ for MFV, 3.06 × 10^−12^ m^2^ s^−1^ for MPPV, 3.01 × 10^−12^ m^2^ s^−1^ for CFV, and 2.96 × 10^−12^ m^2^ s^−1^ for CPPV. The relatively narrow range of values suggests that none of the formulations altered transport by several orders of magnitude; however, the compositional trend was consistent across the calculated values: MFV exhibited the highest D, whereas CPPV showed the lowest.

The MFV value, slightly higher than that of the neat matrix, indicates additional transport pathways in the E-CR/epoxy interfacial regions. Incorporating glass fibers substantially increases the interfacial area. If local discontinuities, microvoids, or poorly wetted regions are present, they may facilitate water ingress and act as preferential pathways. Buckingham et al. [12] and Feng et al. [3] emphasized that GFRP durability in humid environments depends on both matrix permeability and the integrity of the fiber–matrix interface. Water can penetrate this region, modify interfacial interactions, and promote subsequent debonding or interfacial damage.

Increasing the E-CR content from the MFV to the CFV formulation, in contrast, reduced the calculated effective diffusion coefficient from 3.13 × 10^−12^ to 3.01 × 10^−12^ m^2^ s^−1^. This result indicates that, under the specific manufacturing conditions analyzed, increasing the reinforcement content did not produce a proportional increase in rapid interfacial transport pathways. One possible explanation is that a higher fraction of continuous fibers reduces the relative volume of matrix available for transport and simultaneously increases the tortuosity of the path water molecules follow.

Recent studies confirm the intrinsically competitive nature of moisture transport in reinforced materials. Elen et al. [27] reported that fibers can reduce the volume of matrix susceptible to moisture uptake, whereas a degraded interface can produce the opposite effect by facilitating water ingress. Öztürk et al. [28] also found that manufacturing and aging conditions affect diffusion in laminated composites and highlighted the importance of reinforcement distribution and interfacial stability. These findings explain why a higher fiber content does not necessarily result in greater moisture uptake; the final response depends on the balance among matrix replacement, transport tortuosity, and the continuity of interfacial regions.

Incorporating PP further decreased D at both E-CR contents. For the system with lower glass fiber content, the coefficient decreased from 3.13 × 10^−12^ m^2^ s^−1^ in MFV to 3.06 × 10^−12^ m^2^ s^−1^ in MPPV. For the system with the higher glass fiber content, it decreased from 3.01 × 10^−12^ to 2.96 × 10^−12^ m^2^ s^−1^ from CFV to CPPV. This trend is consistent with the gravimetric results shown in Figure 7 and therefore provides converging evidence that E-CR/PP hybridization is associated with lower effective water mobility within the material.

The CPPV formulation is particularly relevant because it simultaneously exhibited the lowest mass gain after 15 days (1.2%) and the lowest effective diffusion coefficient (2.96 × 10^−12^ m^2^ s^−1^). This agreement indicates that the result is not merely an isolated variation in swelling rate, but rather a consistent pattern across two independent parameters derived from the gravimetric test. The combination of 50% E-CR and 5% PP appears to create a structure in which the lower available matrix volume, PP’s low affinity for water, and the greater tortuosity of the transport pathways at least partially counteract moisture ingress through the interfaces.

Wang et al. [7] reported similar behavior in PP-based GFRP materials intended for marine applications. In these systems, moisture absorption exhibited Fickian-type characteristics at low water concentrations, and degradation progression depended strongly on interfacial stability. Rubio-González et al. [4], in a study combining moisture absorption and acoustic emission in seawater-aged glass fiber/epoxy composites, demonstrated that moisture diffusion and damage response are closely related. These findings are particularly relevant to the present study because they indicate that the diffusion coefficient should not be regarded as an independent property, but rather as a variable associated with the subsequent evolution of load transfer and interfacial damage.

The differences among the effective diffusion coefficients are also relatively small. The reduction between the two extreme formulations, MFV and CPPV, is approximately 5.4%. Therefore, although the trend favors CPPV, it would not be appropriate to describe this difference as statistically significant without specific replication and inferential analysis of D.

The results also indicate that the swelling rate after 15 days ranged from 0.031 to 0.034 g day^−1^, whereas the variations in cumulative mass gain were considerably larger. This indicates that composition primarily influenced the total moisture absorbed during the preceding stage, rather than only the instantaneous rate at 15 days. In other words, two formulations may show similar swelling rates at a given time but have different cumulative absorption histories. This distinction is essential to avoid conflating swelling rate with total absorbed water.

In aged polymer systems, this evolution may relate to gradual changes in free volume, matrix relaxation, and transformations within interfacial regions. Mirzapour et al. [29] showed that changes in thermoset matrix structure can alter both moisture uptake and the effective pathways for water ingress. In contrast, Sindu and Sasmal [24] showed that salts can modify epoxy matrix response beyond simple diffusion. Therefore, the observed differences are better attributed to effective moisture transport controlled by multiple phases and interfacial regions rather than solely to homogeneous diffusion through the matrix.

The epoxy matrix initially absorbed moisture rapidly and then gradually approached a stable state. Adding E-CR modifies water transport with reinforcement content, whereas incorporating PP reduced both cumulative mass gain and the diffusion coefficient. Within the compositional range analyzed, CPPV showed the most favorable moisture-transport response. However, gravimetric absorption alone cannot establish the technical significance of this reduction. The diffusion behavior must be supported by demonstrating its relationship to residual mechanical response and damage progression, particularly because deterioration in marine composites generally results from the interaction among matrix plasticization, interfacial transport, and the gradual loss of load-transfer efficiency [3,7,12].

In this sense, the main finding is that E-CR/PP hybridization not only changes the reinforcement content but also modifies the system’s transport structure. The lower moisture uptake and lower estimated D of CPPV provide an experimental basis for determining whether this composition also retains its mechanical properties more effectively after marine exposure. Demonstrating such a relationship would be more meaningful than merely reducing diffusion, because it would enable establishing a structure–transport–damage–property sequence to explain the hybrid composite’s mechanical behavior.

### 3.5. Flexural Testing

The flexural response clearly depended on both material composition and seawater exposure (Figure 8). The neat epoxy resin reached a maximum flexural stress of approximately 480 MPa in dry conditions, but after 30 days of immersion, it decreased to approximately 80 MPa. For the E-CR glass fiber-reinforced systems, dry MFV and CFV reached strengths of approximately 300 and 900 MPa, respectively; after immersion, these values decreased to approximately 200 and 420 MPa, respectively. After marine exposure, the hybrid formulations MPPV and CPPV reached approximately 320 and 600 MPa, respectively. In addition, the report classifies ductility as high for the epoxy matrix, moderate for MFV, low for CFV, moderately high for MPPV, and improved for CPPV.

The high strength of CFV under dry conditions shows that increasing the E-CR content can significantly improve the system’s capacity to withstand flexural stresses. The value of approximately 900 MPa represents an 87.5% increase relative to the 480 MPa of the neat epoxy matrix. This behavior is attributed to the greater contribution of the continuous glass fibers to longitudinal load transfer, particularly in the tensile region during flexure. In a composite with fibers aligned along the principal loading direction, increasing the reinforcement fraction increases the stiffer phase’s contribution to load bearing, provided load transfer across the fiber–matrix interface is sufficiently efficient. This behavior is consistent with current understanding of GFRP, in which flexural strength depends not only on fiber strength but also on impregnation quality, matrix integrity, and interfacial efficiency [3,12].

However, MFV requires more careful interpretation. Although the report states that adding 25% glass fiber increases strength, the experimental MFV value (approximately 300 MPa) is lower than the approximately 480 MPa exhibited by the dry epoxy resin. Therefore, based on the current data, it would not be correct to state that 25% E-CR content increased flexural strength relative to the matrix. This result suggests a reinforcement threshold below which fiber incorporation does not necessarily improve the system’s strength. Possible explanations include interfacial regions, impregnation heterogeneities, or local stress concentrations that offset the fibers’ reinforcing contribution.

Thirty days of exposure to the marine environment caused a marked reduction in maximum stress in all systems for which an equivalent dry condition was available. As shown in Table 4, the epoxy resin decreased by approximately 83.3%, compared with 33.3% for MFV and 53.3% for CFV. In terms of residual strength relative to the initial value, these reductions correspond to approximate retentions of 16.7%, 66.7%, and 46.7%, respectively. The severity of the decrease in the matrix indicates that the epoxy phase is one of the components most sensitive to aqueous conditioning, although the fibers help preserve a greater proportion of the material’s load-bearing capacity.

The reduction in strength after immersion is consistent with aging mechanisms recently described for epoxy matrices and glass fiber-reinforced composites. Absorbed water can increase segmental mobility in the matrix through plasticization, alter its stiffness, and progressively reduce the efficiency of stress transfer at the interface. Wang et al. [7] demonstrated that moisture absorption in polymer matrices exposed to seawater is directly related to changes in their mechanical properties. Yalçınkaya et al. [6] observed that marine exposure modifies the mechanical properties of glass fiber-reinforced composites, whereas Alsaadi et al. [9] associated losses in flexural strength during seawater aging with moisture uptake, matrix response, and progressive deterioration of the interfacial regions. Therefore, the decrease in strength in the E-CR/epoxy systems can be regarded as a combined effect of changes in the matrix and interfacial efficiency rather than degradation in a single phase.

This mechanism is particularly relevant when considering the moisture absorption results previously obtained for the same system. The epoxy matrix showed progressive moisture uptake during immersion, whereas the reinforced formulations exhibited effective diffusion coefficients on the order of 10^−12^ m^2^ s^−1^. The coexistence of moisture transport and strength reduction provides a physical basis linking both responses. Water absorbed by the matrix can act as a plasticizer, while its transport through interfacial regions can gradually weaken stress transfer between the fiber and the resin. José-Trujillo et al. [8] also reported this relationship between diffusion and mechanical deterioration, clearly linking absorbed water content to the evolution of the mechanical properties of seawater-aged GFRP.

Comparison of MFV and CFV also shows that higher initial strength does not necessarily imply better retention after aging. CFV reached the highest maximum stress under dry conditions, approximately 900 MPa, but decreased to about 420 MPa after immersion, corresponding to a 53.3% loss. In contrast, MFV decreased from approximately 300 to 200 MPa, representing a 33.3% loss. Thus, although CFV retained a higher absolute strength than MFV after exposure, its relative loss was greater. This behavior may be associated with the multiphase nature of damage in composites with high reinforcement contents. As fiber content increases, the total fiber–matrix interfacial area also increases; if part of this region becomes susceptible to water ingress, higher reinforcement content may increase initial load-bearing capacity while enlarging regions vulnerable to interfacial degradation.

This interpretation is like recent research on GFRP durability in marine settings, showing that residual performance depends on the interplay between fiber strength, matrix stability, and interfacial integrity [3,12]. Rubio-González et al. [4] demonstrated that seawater aging of glass fiber/epoxy systems modifies both flexural strength and damage progression, as detected by acoustic emission, highlighting that the loss of macroscopic load-bearing capacity is closely linked to changes in internal damage mechanisms. Ghabezi and Harrison [2] identified interfacial debonding as a key mechanism governing the response of composites aged in saline environments.

The response of hybrid formulations represents one of the most relevant findings of this study. After immersion, MPPV reached approximately 320 MPa, compared with about 200 MPa for immersed MFV. Likewise, CPPV reached nearly 600 MPa, compared with approximately 420 MPa for immersed CFV. Considering only the immersed materials, MPPV exhibited approximately 60% higher strength than MFV, whereas CPPV exceeded CFV by about 42.9%. These results suggest that PP incorporation enhances the residual mechanical response of the E-CR/epoxy systems under the conditions investigated.

This effect should not be interpreted simply as an additional strength contribution from the PP fibers. Because PP fibers are less stiff than glass fibers, their role in the hybrid system likely involves more complex changes in damage evolution and composite deformability. MPPV exhibited moderately high ductility, whereas CPPV was more ductile than CFV, which showed low ductility. Incorporating a more deformable secondary fibrous phase may facilitate stress redistribution after damage initiation in the matrix or at the E-CR/epoxy interfaces, thereby delaying localization of critical strain. This mechanism is consistent with the hybridization principle in multifiber composites, in which a stiff phase primarily carries the load while a more deformable phase improves damage tolerance.

The previous moisture absorption results support an additional mechanism. The PP-containing formulations exhibited lower mass gain than their counterparts with the same E-CR content, and CPPV showed the lowest estimated effective diffusion coefficient. The relationship between lower moisture uptake and higher residual flexural strength indicates a structure–transport–property interaction: PP may alter water-transport pathways and reduce the fraction of matrix available for direct interaction with moisture, while its greater deformability may help redistribute stresses once the matrix and interfacial regions are affected. However, the current results do not allow quantification of each mechanism’s contribution.

The CPPV formulation is particularly noteworthy in this context. After immersion, it exhibited a residual maximum stress of approximately 600 MPa, higher than that of all other marine-exposed formulations. In addition, the report notes that it exhibited a more favorable combination of strength and ductility. This behavior is consistent with recent studies on PP-based GFRP systems. Wang et al. [7] showed that moisture ingress and changes at the fiber–matrix interface primarily influence the stability of glass/PP materials in marine environments. Kumar et al. [30] observed that glass fiber-reinforced thermoplastic systems may age differently than thermosetting systems because of differences in moisture absorption, matrix structure, and load-transfer mechanisms. These findings support the hypothesis that adding PP may improve the hybrid system’s response after water exposure.

The high sensitivity of epoxy resins to water absorption is well documented; therefore, the apparent 83.3% loss in only 30 days should be discussed, given that the dry and immersed specimens did not share the same curing history. Wang et al. [7] showed that the curing state can modify both moisture uptake and the residual properties of a polymer matrix, while studies on hygrothermal aging have identified the degree of crosslinking as a key variable governing water resistance.

These comparisons refer to residual flexural strength after the same 30-day seawater exposure, not strength retention relative to the dry state. Strength retention requires normalizing the post-exposure strength to the corresponding dry-state value for the same formulation. Because equivalent dry-state baseline data are not available for MPPV and CPPV, strength-retention percentages cannot be determined for these hybrid formulations. Moreover, the dry-state values of MFV and CFV cannot be used as surrogate baselines because the incorporation of 5 wt.% PP modifies both the constituent fractions and the reinforcement architecture. Therefore, the present results demonstrate higher residual flexural strength of the PP-containing formulations after 30 days of seawater immersion, but they do not establish improved flexural-strength retention relative to their initial dry state.

The results indicate that the system’s flexural response depends on the interaction among E-CR content, PP incorporation, and environmental conditions. Increasing E-CR content to 50% produced the highest strength under dry conditions, but it also showed a substantial relative loss after conditioning. In contrast, PP incorporation led to higher residual strength and a qualitative improvement in the ductility of the exposed materials. CPPV reached approximately 600 MPa after 30 days of immersion, compared with about 420 MPa for CFV under the same conditions. In addition, previous gravimetric results showed that CPPV absorbed less moisture and had a lower estimated effective diffusion coefficient. This convergence indicates that E-CR/PP hybridization modifies both moisture transport and damage resistance, resulting in a more favorable balance between load-bearing capacity and residual deformability.

### 3.6. Micromechanical Tension Testing and Acoustic Emission

Samples for both characterizations, the Hsu-Nielsen test and the tensile mechanical test, were manufactured by embedding a single filament within the epoxy matrix, as shown in Figure 2 in Section 2.5. The micromechanical response indicates that adding a single filament affects elastic wave transmission and the evolution of acoustic activity during loading differently. The Hsu-Nielsen test yielded propagation velocities of 201.13 ± 10.55 m s^−1^ for the epoxy matrix, 353.30 ± 18.54 m s^−1^ for the epoxy/E-CR system, and 207.03 ± 8.94 m s^−1^ for epoxy/PP. In relative terms, the E-CR filament increased the propagation velocity by approximately 75.7% compared with the matrix, whereas adding PP increased it by only 2.9%. This difference is significant because it shows that the two reinforcement types affect the system’s effective dynamic response in clearly distinct ways.

The maximum velocity observed in the E-CR/epoxy system indicates more efficient transmission of elastic disturbance through the composite. However, this response cannot be attributed solely to interfacial adhesion quality, because propagation velocity also depends on phase stiffness, continuity, specimen geometry, fiber–matrix coupling, and discontinuities. In this sense, the difference between E-CR/epoxy and PP/epoxy can be attributed to the stiff glass phase and a potentially more effective interface in the E-CR system. In contrast, PP, because of its lower stiffness and lower surface polarity, produces a much more limited change in the effective propagation velocity. Recent literature on interface engineering in polymer composites indicates that micromechanical response depends on the combined effects of surface chemistry, wettability, roughness, and the elastic properties of the constituent phases rather than on a single factor [16,31].

This interpretation is similar to previous fiber characterizations. In E-CR, the material includes a silane-based sizing system intended to improve compatibility between the inorganic glass surface and the organic matrix. In contrast, PP has predominantly nonpolar surface chemistry, which limits its ability to interact with a polar epoxy matrix. The report indicates that PP’s greater surface roughness, as detected by AFM, may promote a predominantly physical or mechanical interlocking mechanism. This explanation agrees with recent studies indicating that silane coupling agents can enhance stress transfer at glass/epoxy interfaces. However, for unmodified polyolefin surfaces, adhesion relies primarily on surface topography and physical contact [16,31].

The mechanical response, combined with acoustic emissions, provides additional information on damage progression as presented in Figure 9. The epoxy matrix reached a maximum stress of about 70 MPa and a strain of about 7%, with acoustic events occurring near the peak stress. The signals showed amplitudes near 60 dB and energies of about 6–8 mV, while the overall range recorded during the test was 50–100 dB and 1–9 mV. The concentration of acoustic activity near the maximum stress indicates that internal discontinuities form and grow as the matrix approaches its strength limit. This pattern is consistent with microdamage nucleation and propagation in thermosetting materials under tensile loading.

The epoxy resin/E-CR fiber composite exhibited a different mechanical response. It reached a maximum stress of approximately 42 MPa and a strain of about 13%, representing a reduction in maximum stress relative to the epoxy matrix together with a substantial increase in ultimate strain. Acoustic signals near the maximum stress showed amplitudes of 55–70 dB and energies of 1–5 mV. The lower maximum stress and higher strain indicate that a single filament changes how the specimen redistributes load and develops damage. The increase in deformability should not automatically be interpreted as conventional mechanical reinforcement because, in a single-filament geometry, filament position, interfacial integrity, and damage initiation in the surrounding matrix strongly influence the overall response.

Literature reports indicate that acoustic events in the 55–70 dB range are associated with fiber fracture, suggesting efficient stress transfer. This hypothesis is mechanically plausible: when the interface transfers load from the matrix to the fiber, the filament may locally reach its critical strength and fracture [22,32,33].

The difference between the Hsu-Nielsen propagation velocities and the mechanical-acoustic response also provides an important observation: the E-CR/epoxy system exhibited the highest propagation velocity, although it did not show the highest tensile strength in the single-filament specimen. This indicates that higher acoustic velocity cannot directly substitute for mechanical strength. Propagation velocity reflects the medium’s ability to transmit elastic disturbances and is governed primarily by effective stiffness and internal continuity, whereas maximum strength depends on how critical damage nucleates and propagates. Therefore, the two parameters are complementary: propagation velocity provides information on the medium’s overall elastic response, whereas acoustic emission during loading enables monitoring of the initiation and evolution of internal discontinuities.

In PP, the propagation velocity of 207.03 ± 8.94 m s^−1^ was nearly identical to that of the epoxy resin, indicating that adding a single PP filament has a limited effect on the specimen’s effective dynamic stiffness. This behavior is consistent with PP’s lower intrinsic stiffness and an interface where load transfer is likely governed more by physical interlocking than by strong chemical interactions. However, PP does not significantly increase propagation velocity, which does not mean its effect on hybrid composite behavior is negligible. Its greater deformability may facilitate stress redistribution mechanisms and enhance damage tolerance at larger scales, particularly when combined with the stiffer E-CR phase. This possibility becomes especially relevant when considering the macroscopic response of the hybrid composites, in which the PP-containing formulations exhibited a favorable residual response after immersion.

The micromechanical results show that E-CR and PP generate mechanically distinct interfacial environments within the epoxy matrix. E-CR produces a marked increase in propagation velocity and generates an acoustic response concentrated near the point of maximum stress, suggesting more efficient load transfer to a stiff phase. In contrast, PP produces only a minor change in the medium’s effective propagation velocity, consistent with different interfacial and elastic contributions. The presence of both reinforcements in the hybrid material may therefore produce a complementary response; E-CR provides stiffness and load-transfer capability, whereas PP may assist in strain redistribution and in controlling damage localization. This interpretation establishes a mechanistic link between the microscale response and the macroscopic behavior of the hybrid composites, although it should be regarded as a hypothesis supported by converging evidence rather than as direct proof of the interfacial mechanisms.

These results reflect the combined contribution of mechanical response and acoustic emission. While the stress–strain curves indicate when the system’s load-bearing capacity changes, AE helps identify the onset of internal damage accumulation. Recent studies indicate that this combination improves damage interpretation in FRP, particularly when multiple acoustic parameters are used rather than amplitude alone [7,33,34]. Reinforcement affects both wave transmission and the development of acoustic activity, although it does not clearly establish which fracture mechanism corresponds to each amplitude range.

The E-CR/epoxy interface shows a distinct acoustic-mechanical response compared with PP/epoxy and the neat matrix, particularly in increased propagation velocity and the behavior of acoustic events near maximum stress. The PP-containing system’s response is closer to that of the matrix in terms of propagation velocity, supporting a different interfacial mechanism. Integration of these results with AFM, FTIR, and the macroscopic response of the composites suggests that E-CR/PP hybridization combines two distinct types of interaction with the matrix: one characterized by higher stiffness and greater chemical compatibility in E-CR/epoxy, and another governed primarily by physical interaction, surface roughness, and deformability in PP/epoxy. This contrast provides a physicochemical and micromechanical basis for interpreting how hybrid composition may influence load transfer and damage development.

A limitation of the present study is the absence of systematic post-fracture microscopic characterization of the flexural specimens after seawater aging. Therefore, although the combined moisture-absorption, mechanical, and acoustic-emission results provide complementary evidence of damage evolution, the available experimental evidence cannot conclusively identify specific microscopic failure mechanisms, such as matrix cracking, fiber–matrix debonding, interfacial sliding, and fiber pull-out. Accordingly, these mechanisms should be regarded as interpretations consistent with the observed macroscopic and acoustic responses rather than as directly demonstrated fracture processes. Future work should incorporate SEM fractography of both dry and seawater-aged specimens to directly correlate fracture-surface features with moisture uptake, residual mechanical performance, and acoustic-emission behavior.

## 4. Conclusions

This study investigated the effect of E-CR glass fiber/PP hybridization on the moisture transport, mechanical, and acoustic response of epoxy composites subjected to seawater exposure. By combining FTIR-ATR, AFM, micromechanical characterization, moisture-absorption and diffusion analysis, flexural testing, and acoustic emission, the study demonstrated that composite performance depends not only on reinforcement content but also on the distinct interfacial characteristics introduced by E-CR glass and PP fibers. The main conclusions are summarized as follows:

The study identified distinct reinforcement surfaces and interfacial environments. FTIR-ATR and AFM showed that E-CR glass fibers and PP fibers present different chemical and topographical characteristics. E-CR glass exhibited contributions from the inorganic glass surface and its commercial sizing, whereas PP showed hydrocarbon characteristics and greater topographical heterogeneity predominantly. These differences provide distinct interfacial environments within the epoxy matrix.

Seawater exposure significantly affected the neat epoxy matrix. Moisture uptake increased progressively during immersion, reaching approximately 4.28% after 364 days. The simultaneous 22.03% reduction in acoustic-wave propagation velocity indicates a substantial change in the effective elastic response of the matrix, consistent with moisture-induced plasticization and reduced effective stiffness.

PP incorporation modified the short-term moisture-transport behavior of the E-CR/epoxy composites. At equivalent E-CR contents, the PP-containing formulations showed lower moisture uptake than their non-hybrid counterparts. Among the investigated composites, CPPV (50 wt.% E-CR + 5 wt.% PP) exhibited the lowest measured moisture uptake (1.20%) and the lowest estimated effective diffusion coefficient (2.96 × 10^−12^ m^2^ s^−1^) during the evaluated immersion period.

The hybrid formulation containing 50 wt.% E-CR and 5 wt.% PP exhibited the most favorable residual flexural response after seawater conditioning. After 30 days of immersion, CPPV showed a residual flexural strength of approximately 600 MPa, compared with approximately 420 MPa for CFV with the same E-CR content but without PP. This result indicates that incorporating PP influenced the composite’s mechanical response after conditioning.

Micromechanical and acoustic measurements revealed different responses for the E-CR/epoxy and PP/epoxy systems. These differences indicate that the two reinforcement types interact differently with the epoxy matrix and affect stress transfer and acoustic-wave propagation in distinct ways. However, acoustic-emission amplitude alone cannot conclusively assign specific microscopic failure mechanisms.

E-CR/PP hybridization should therefore not be considered simply as the addition of a secondary reinforcing fiber. Within the 30-day marine-aging period investigated, the combined experimental evidence indicates that PP incorporation modifies the effective moisture-transport environment and mechanical response of E-CR/epoxy composites. Nevertheless, longer-term aging and post-fracture microscopic characterization are required to determine whether these advantages persist during prolonged seawater exposure and to directly identify the associated damage mechanisms.

## Figures and Tables

**Figure 1:**
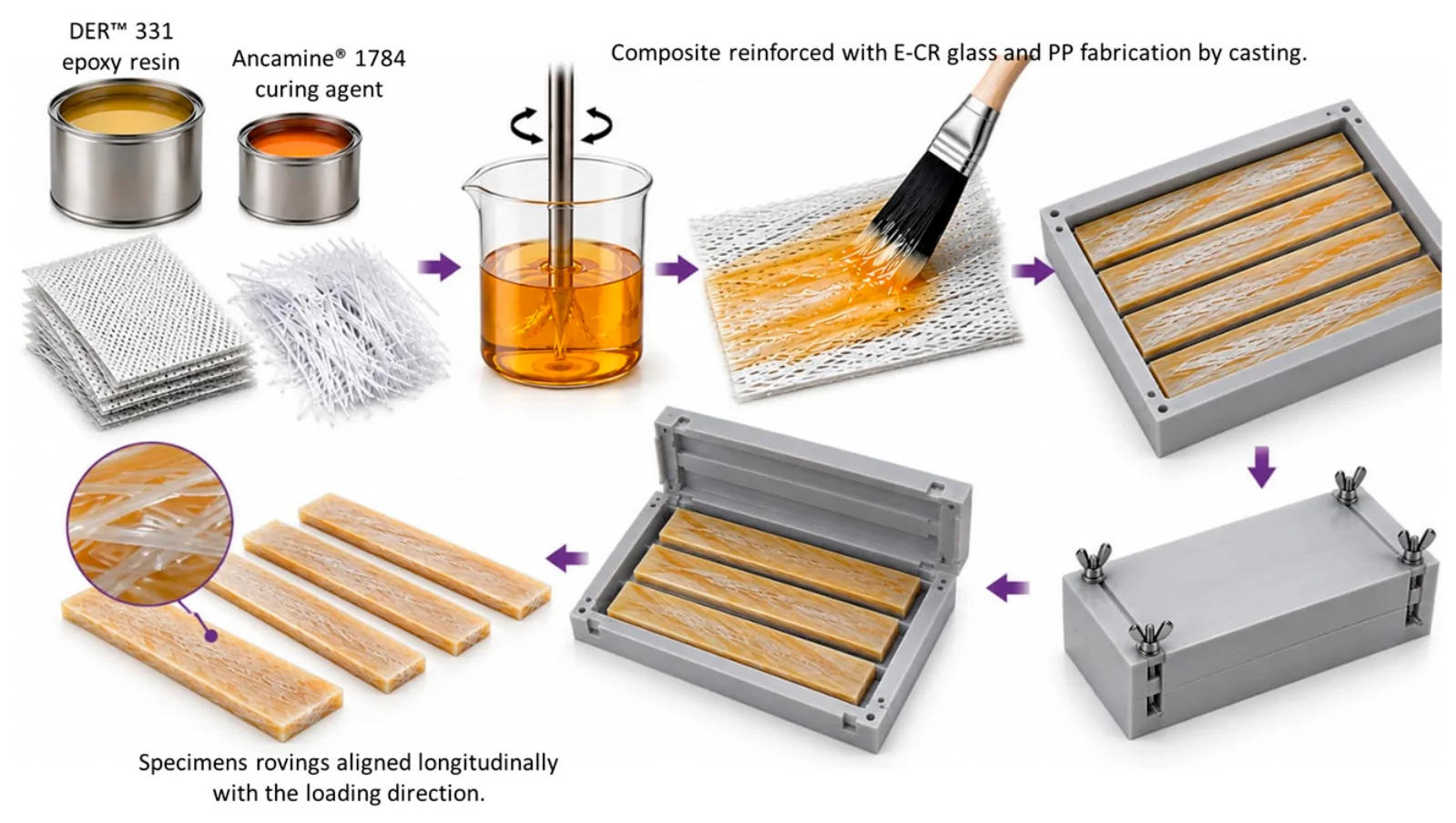
Schematic illustration of the manufacturing process of polymer composite.

**Figure 2:**
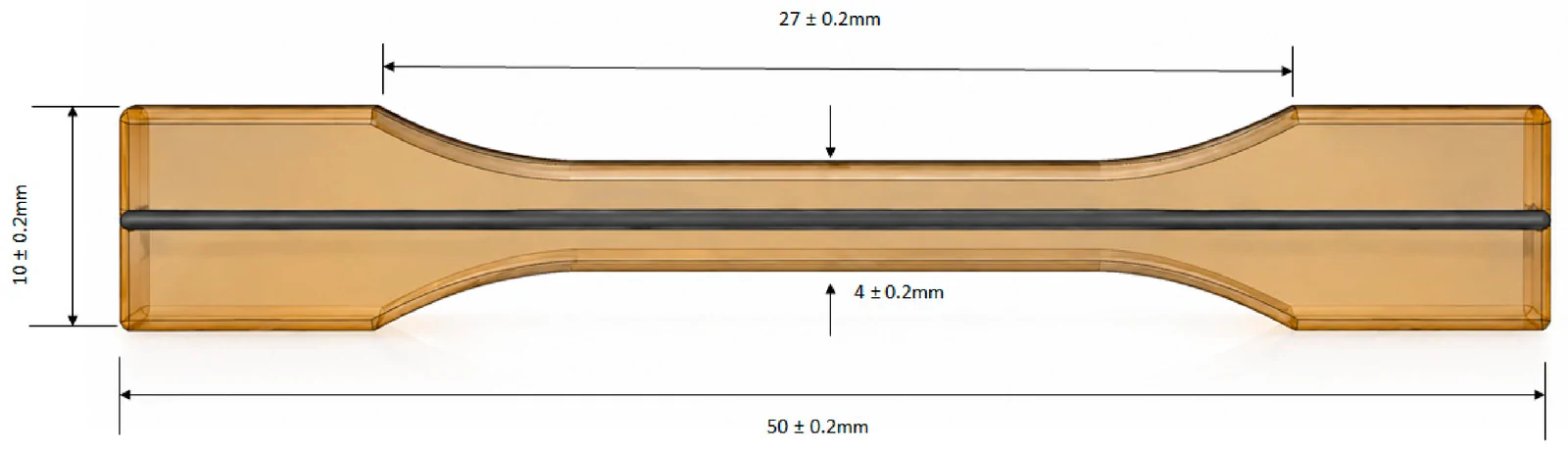
Dog-bone specimen for the Hsu-Nielsen and tensile mechanical characterizations.

**Figure 3:**
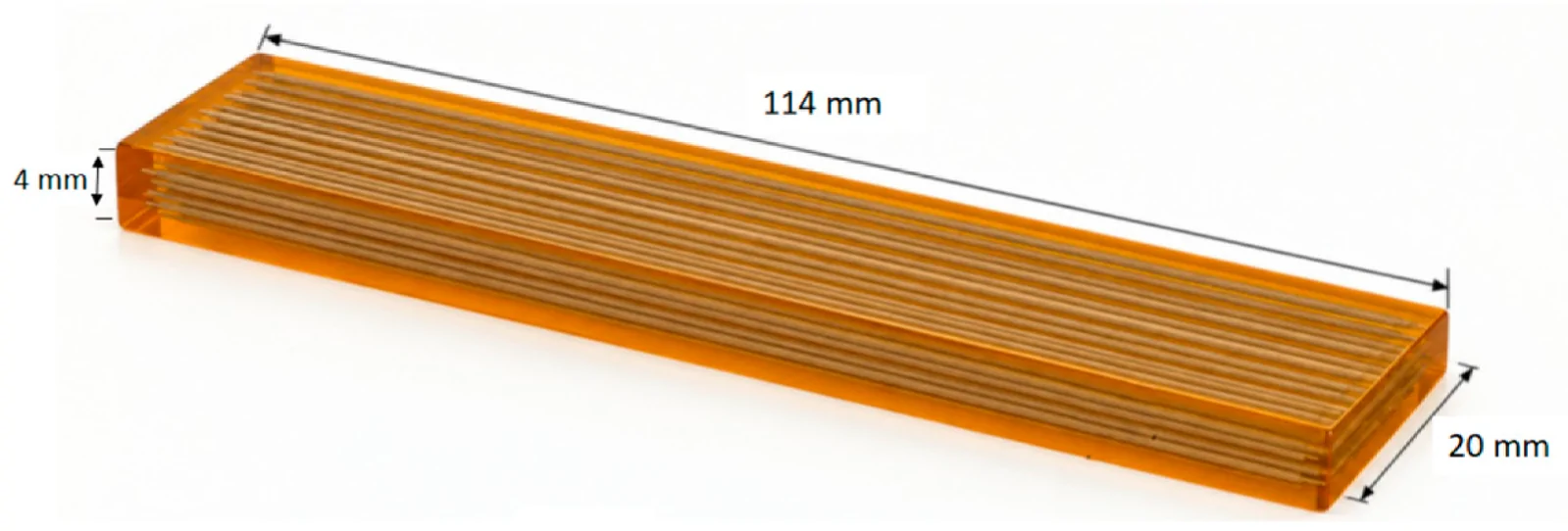
Samples for flexural testing in dried and water-exposed conditions.

**Figure 4:**
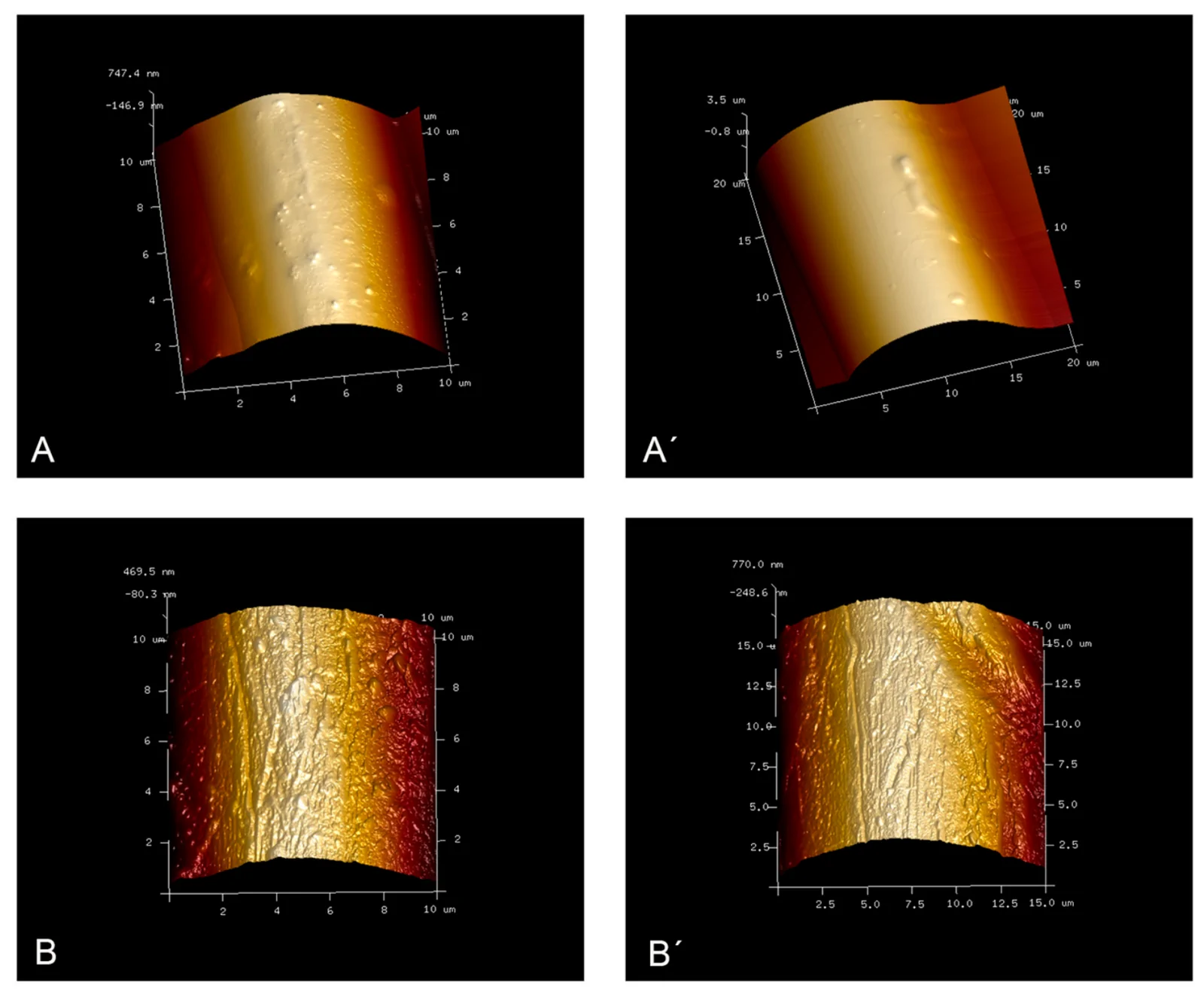
Analysis of fiber surfaces using AFM: average roughness of (**A**) glass fiber filament sample one; (**A′**) glass fiber filament sample two; and (**B**) polypropylene filament sample one; (**B′**) polypropylene filament sample two.

**Figure 5:**
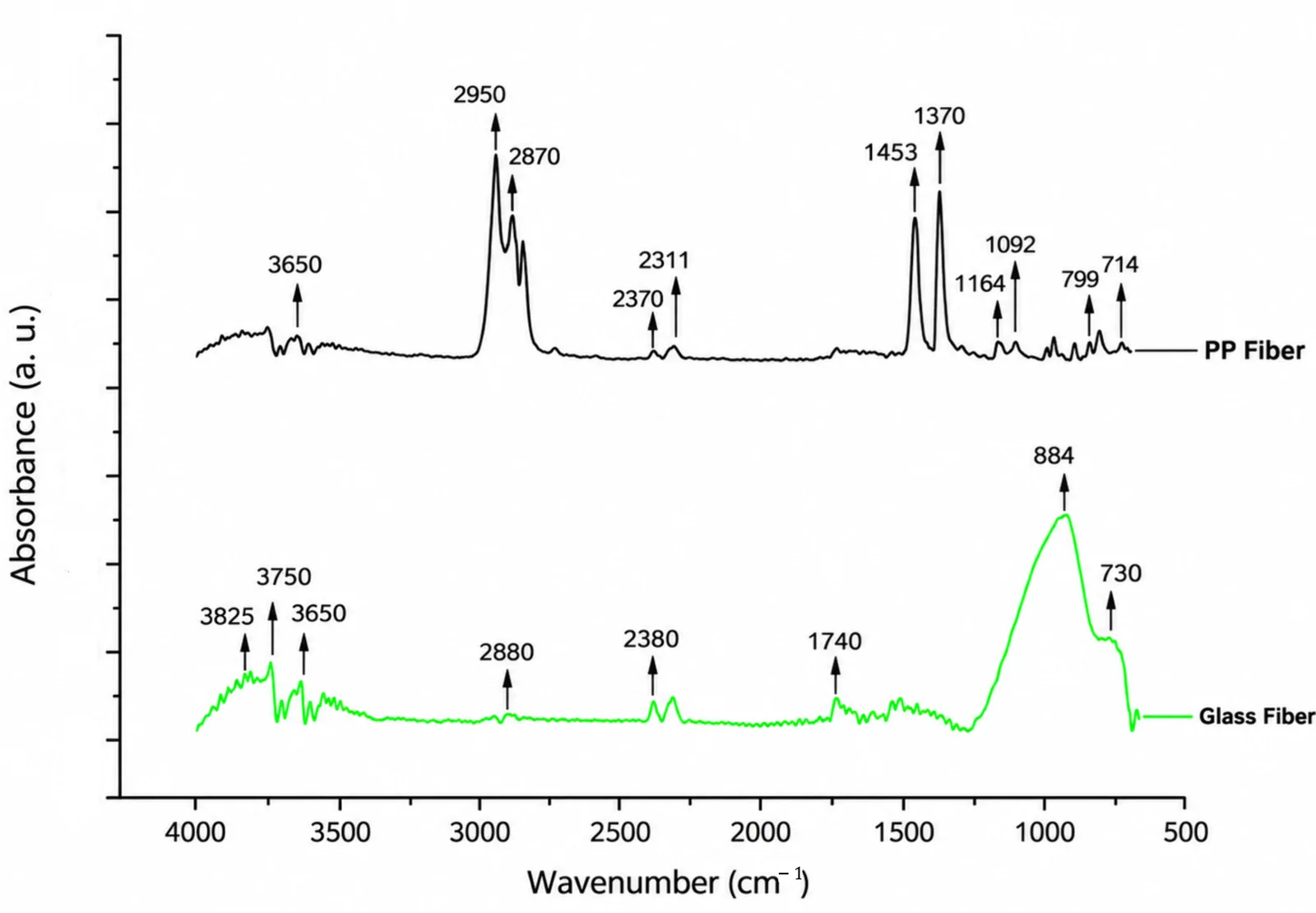
FTIR spectrum of polypropylene and glass fibers.

**Figure 6:**
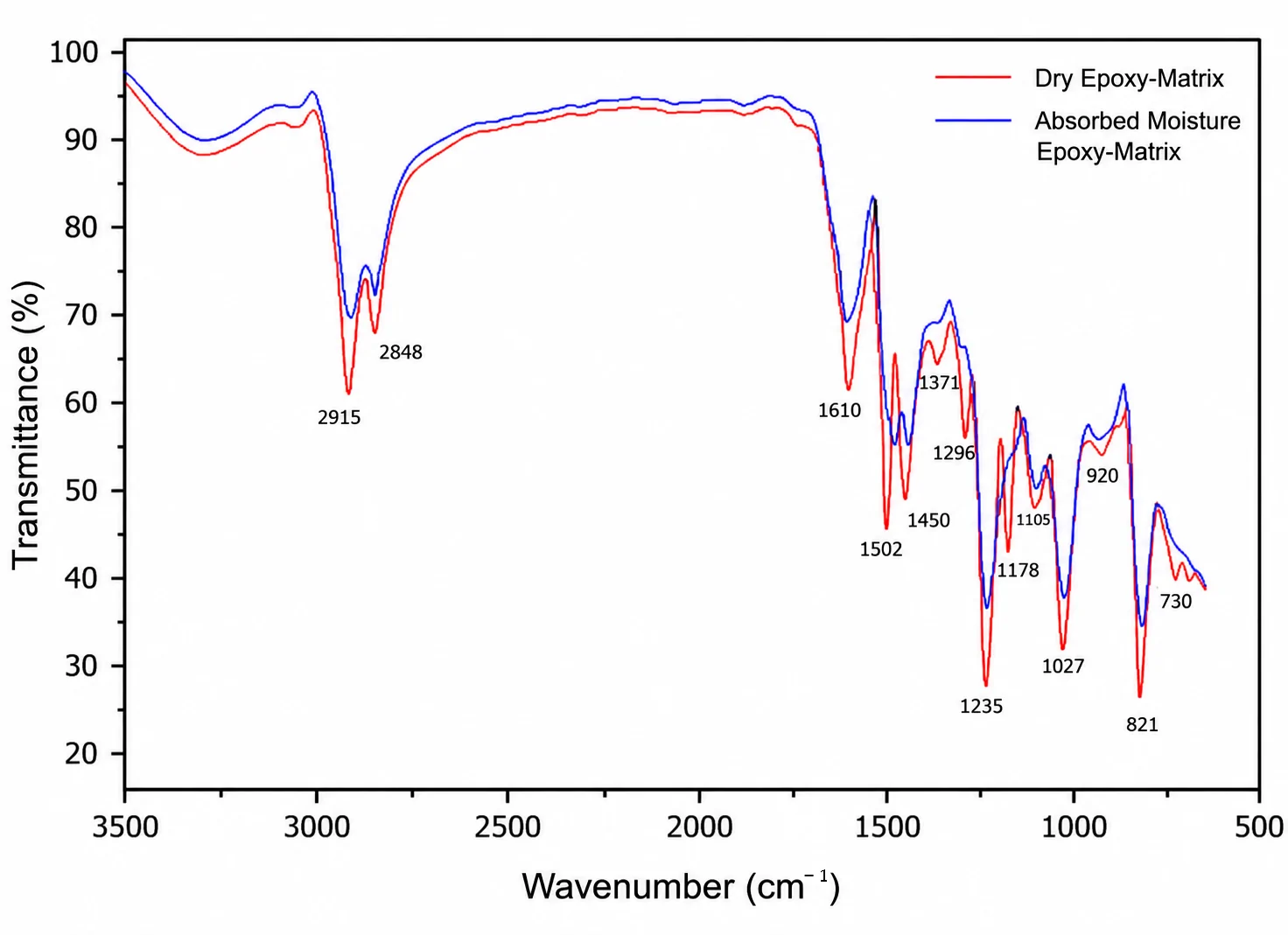
FTIR spectrum of epoxy resin before and after 24 days of immersion in saltwater.

**Figure 7:**
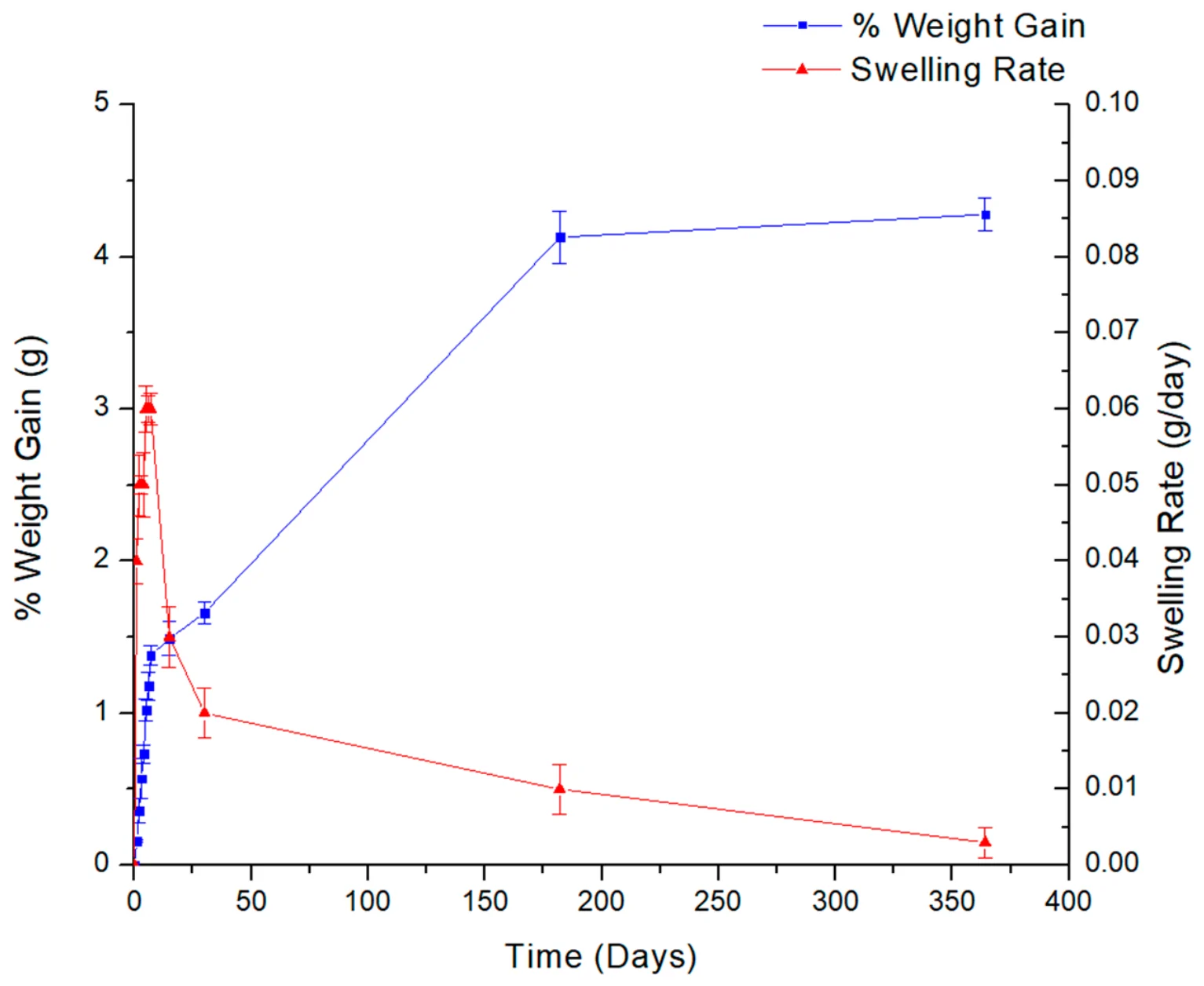
Absorption and swelling rate of the epoxy specimens (without fibers).

**Figure 8:**
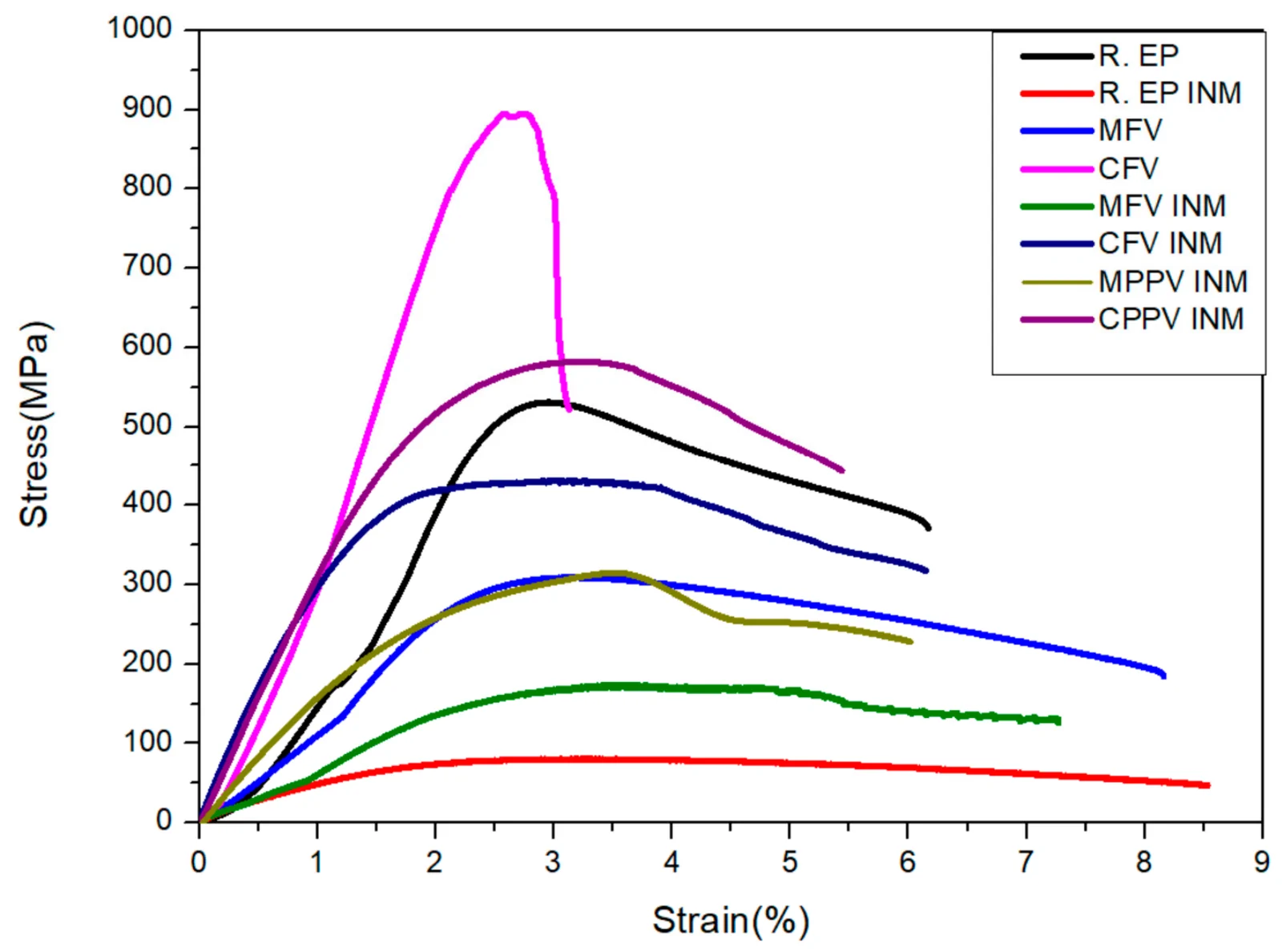
Flexural stress–strain curves for various epoxy resins with reinforcements.

**Figure 9:**
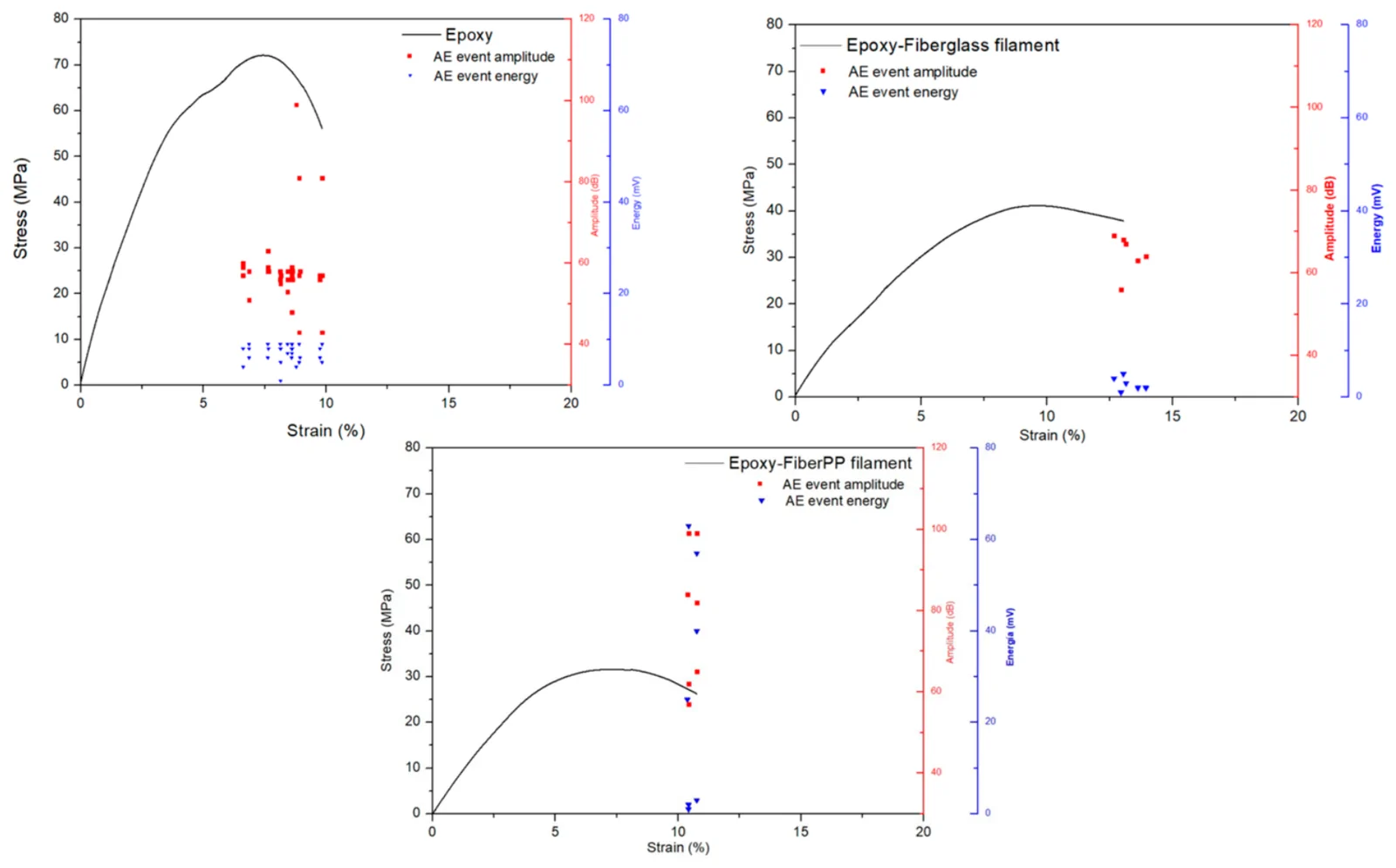
Stress–strain curves for epoxy resin, epoxy resin/glass fiber filament, and epoxy resin/polypropylene fiber filament specimens, incorporating the acoustic emission technique to measure the amplitude and energy emitted during tensile mechanical characterization.

**Table 1:** Final mass fractions of the constituents.

Formulation	Epoxy Matrix (wt.%)	E-CR Glass Fiber (wt.%)	PP Fiber (wt.%)	Total (wt.%)
Epoxy	100	0	0	100
MFV	75	25	0	100
MPPV	70	25	5	100
CFV	50	50	0	100
CPPV	45	50	5	100

Note: MFV = epoxy composite containing 25 wt.% E-CR glass fiber; MPPV = hybrid epoxy composite containing 25 wt.% E-CR glass fiber and 5 wt.% PP fiber; CFV = epoxy composite containing 50 wt.% E-CR glass fiber; CPPV = hybrid epoxy composite containing 50 wt.% E-CR glass fiber and 5 wt.% PP fiber.

**Table 2:** Gravimetric evidence and acoustic effect of water incorporated into the epoxy matrix.

Sample	Weight (g)	Sound Wave Velocity (m s^−1^)
Without immersion	25.2	1645.16
With immersion	25.7	1282.79

**Table 3:** Diffusion coefficient calculation after 15 days of immersion in seawater.

Sample	D (m^2^ s^−1^)
Blank	3.04 × 10^−12^
MFV	3.13 × 10^−12^
MPPV	3.06 × 10^−12^
CFV	3.01 × 10^−12^
CPPV	2.96 × 10^−12^

**Table 4:** Effect of seawater on epoxy resin and composites’ mechanical parameters.

Sample	Max. Strength (MPa)	Elastic Modulus (GPa)	Strain to Failure (%)
Epoxy resin	530.93 ± 25.88	2.27 ± 1.88	6.16 ± 0.43
Epoxy resin immersed in seawater	80.15 ± 16.52	1.31 ± 0.83	8.52 ± 0.33
MFV: Epoxy resin + 25% glass fiber.	305.21 ± 13.41	3.01 ± 1.19	8.16 ± 0.32
MFV immersed in seawater	172.85 ± 12.10	2.67 ± 1.06	7.26 ± 0.43
CFV: Epoxy resin + 50% glass fiber.	894.6 ± 25.48	5.25 ± 3.01	3.57 ± 0.88
CFV immersed in seawater	417.12 ± 20.8	3.57 ± 2.58	6.14 ± 0.88
MPPV: MFV + 5% polypropylene, immersed in seawater	314.99 ± 16.87	4.01 ± 0.58	6.01 ± 0.35
CPPV: CFV + 5% polypropylene, immersed in seawater	581.13 ± 18.58	3.68 ± 0.48	5.44 ± 0.16

## Data Availability

All data generated or analyzed during this study are included in this published article.
